# Potential responders to FOLFOX therapy for colorectal cancer by Random Forests analysis

**DOI:** 10.1038/bjc.2011.505

**Published:** 2011-11-17

**Authors:** S Tsuji, Y Midorikawa, T Takahashi, K Yagi, T Takayama, K Yoshida, Y Sugiyama, H Aburatani

**Affiliations:** 1 Genome Science Division, Research Center for Advanced Science and Technology, The University of Tokyo, Tokyo, Japan; 2 Department of Surgery, Teikyo University School of Medicine University Hospital, Mizonokuchi, Kawasaki, Japan; 3 Department of Digestive Surgery, Nihon University School of Medicine, Tokyo, Japan; 4 Department of Oncologic Surgery, Gifu University, Gifu, Japan

**Keywords:** colorectal cancer, FOLFOX therapy, machine learning algorithm, class predictor, personalised therapy

## Abstract

**Background:**

Molecular characterisation using gene-expression profiling will undoubtedly improve the prediction of treatment responses, and ultimately, the clinical outcome of cancer patients.

**Methods:**

To establish the procedures to identify responders to FOLFOX therapy, 83 colorectal cancer (CRC) patients including 42 responders and 41 non-responders were divided into training (54 patients) and test (29 patients) sets. Using Random Forests (RF) algorithm in the training set, predictor genes for FOLFOX therapy were identified, which were applied to test samples and sensitivity, specificity, and out-of-bag classification accuracy were calculated.

**Results:**

In the training set, 22 of 27 responders (81.4% sensitivity) and 23 of 27 non-responders (85.1% specificity) were correctly classified. To improve the prediction model, we removed the outliers determined by RF, and the model could correctly classify 21 of 23 responders (91.3%) and 22 of 23 non-responders (95.6%) in the training set, and 80.0% sensitivity and 92.8% specificity, with an accuracy of 69.2% in 29 independent test samples.

**Conclusion:**

Random Forests on gene-expression data for CRC patients was effectively able to stratify responders to FOLFOX therapy with high accuracy, and use of pharmacogenomics in anticancer therapy is the first step in planning personalised therapy.

Combinations of oxaliplatin (OHP) combined with various schedules of antimetabolite 5-fluorouracil (5-FU) and leucovorin (LV) are currently the first-line treatments for unresectable colorectal cancer (CRC), and the superiority of the FOLFOX4 regimen has been demonstrated (de Gramont *et al*, 2000; Giacchetti *et al*, 2000). A much higher response rate was observed (>50%) with the FOLFOX4 regimen than with 5-FU+LV, and this resulted in survival benefits for patients undergoing FOLFOX4 therapy (de Gramont *et al*, 2000; Giacchetti *et al*, 2000), and the modified FOLFOX6 (mFOLFOX6) regimen subsequently showed equivalent efficacy and tolerance (Cheeseman *et al*, 2002; Braun *et al*, 2003). Thus, some patients receive the benefits of FOLFOX therapy, while others undergo ineffective chemotherapy for several cycles until the effects are determined, which can often result in detrimental, life-threatening side effects. Stratification of patients for multidrug response based on biological characteristics is thus indispensable for personalised therapy.

Drug sensitivity in chemotherapy is thought to be attributable to variations in underlying genetic characteristics of cancer. Biomarkers were originally used to measure disease progression and as surrogates of treatment efficacy. However, biomarkers can also be used as predictive markers to indicate whether a patient is a good candidate for a specific drug or regimen (Torri *et al*, 1992; Simon, 2008).

Gene-expression signatures have a great potential both for predicting outcomes in cancer patients, and for predicting response or toxicity with various anticancer drugs (van de Vijver *et al*, 2002; Gordon *et al*, 2003; Nutt *et al*, 2003; Berchuck *et al*, 2005), and they may be superior to conventional clinical and pathological approaches (Parissenti *et al*, 2007). Using gene-expression profiles, Dressman *et al* (2007) were able to identify advanced ovarian cancer patients who were likely to be resistant to platinum-based chemotherapy with more than 80% accuracy. On the other hand, a classifier gene set selected by diagonal linear discriminant analysis predicted pathologic complete response (CR) to paclitaxel and fluorouracil–doxorubicin–cyclophosphamide chemotherapy for breast cancer with high sensitivity in independent cases (Hess *et al*, 2006). When compared with the estimation of responders to anticancer drugs using expression profiling in breast or ovarian cancers, only a small number of such studies have been performed in CRC (Nannini *et al*, 2009). Del Rio *et al* (2007) determined 14 classifier genes for predicting response to combined therapy with LV, 5-FU, and irinotecan (FOLFIRI), although they studied a relatively small number of patients.

In order to maximise the benefits of microarray technology, researchers have attempted to develop several classification algorithms in a reproducible manner (Wu *et al*, 2003; Statnikov *et al*, 2008; Kim *et al*, 2009). Random Forests (RF) is a machine learning algorithm that uses an ensemble of classification trees and is available for microarray data analysis in which the number of variables is much larger than samples (Breiman, 2001). Random Forests was demonstrated to be a part of the standard method for class prediction and gene selection with microarray data (Diaz-Uriarte and Alvarez de Andres, 2006). In addition to this, RF was able to cluster samples; thus, we applied RF to predicting the efficacy of FOLFOX therapy. Some reports have demonstrated that RF, as with other methods including support vector machines (SVMs) and linear discriminant analysis, outperforms in the classification of several cancers (Wu *et al*, 2003; Statnikov *et al*, 2008; Kim *et al*, 2009). Clinically, using RF, tumour class discovery of renal cell carcinoma based on tissue microarray profiling is possible, and this could not be explained by clinicopathological variables (Shi *et al*, 2005).

In the present study, we used the RF algorithm to identify classifier genes that are able to predict responders to FOLFOX therapy for unresectable CRC. Using these biomarkers, we were able to predict the patients most likely to benefit from selected multiagent chemotherapy with a high degree of accuracy. Our results demonstrated that pharmacogenomic identification of predictor genes for response to chemotherapy will benefit advanced cancer patients, which will aid in the development of personalised therapy.

## Materials and Methods

### Patients and tissue samples

A total of 83 patients with unresectable CRC undergoing FOLFOX therapy from April 2007 to December 2010 in Teikyo University Hospital at Mizonokuchi and Gifu University Hospital were recruited in this study. All CRC samples were obtained before mFOLFOX6 therapy, including 56 primary CRCs and 27 metastatic lesions to the liver (23 tumours), lung (1 tumour) and peritoneum (3 tumours). None of the patients enrolled in this study underwent any chemotherapy or radiotherapy in advance. Samples were divided (approximately 2 : 1 ratio) into training and test sets. As a result, 54 of 83 samples obtained in the first half of this period were selected for the training set, and the remaining 29 samples in the latter half were selected as the test set. All subjects gave their informed consent for participation in the study. Clinical parameters and tumour status based on histological findings of resected specimens are summarised in Supplementary Tables S1 and S2.

Surgical specimens were immediately cut into small pieces after resection, snap frozen in liquid nitrogen, and stored at −80 °C.

### Chemotherapeutic regimen and monitoring of FOLFOX therapy

All patients were treated with mFOLFOX6, as proposed by Cheeseman *et al* (2002); 85 mg m^−2^ OHP, 200 mg m^−2^ LV, and 400 mg m^−2^ 5-FU bolus on day 1, and 2400 mg m^−2^ 5-FU as a 46-h continuous infusion starting on day 1, which is repeated every 2 weeks. After four cycles of mFOLFOX6 therapy, all lesions were assessed by computed tomography, and classified as CR (disappearance of all target lesions), partial response (PR, at least a 10% decrease in the sum of the longest diameter of target lesions), progressive disease (PD, at least a 10% increase in the sum of the longest diameter of target lesions), and stable disease (SD, neither sufficient shrinkage to qualify for PR nor sufficient increase to qualify for PD), according to the Response Evaluation Criteria in Solid Tumors (Therasse *et al*, 2000), with minor modification.

### RNA extraction and oligonucleotide microarray for gene-expression studies

After trimming the frozen sample, in order to contain more than 50% tumour nuclei (Yamamoto *et al*, 2010), total RNA was isolated from tumour samples, as described previously (Midorikawa *et al*, 2003). Experimental procedures for GeneChip were performed in accordance with the GeneChip Expression Analysis Technical Manual (Affymetrix, Santa Clara, CA, USA), using 3 *μ*g of total RNA. These data are available at NCBI with GEO Accession no. GSE28702.

### Normalisation and filtering of gene-expression data

Before further statistical analysis, we normalised and filtered the raw data. The Affymetrix Power Tools (Affymetrix. *Technical notes*. Affymetrix, 2005) was used to summarise the probe intensity of the CEL file by apt-probeset-summarise command with plier-mm-skech option. We filtered out the poorly expressed probe sets from the raw data using Gene Pattern (Reich *et al*, 2006) with the following parameter settings: minchange=3, mindelta=100, threshold=20, ceiling=20 000, and num excl=2.

### Random Forests analysis

The RF algorithm was applied to raw data for tumour samples obtained from expression arrays, as described previously (Breiman, 2001). Briefly, RF is a machine-learning algorithm that builds a class prediction model using class labelled input samples and calculates a ranking of input variables ordered by the extent of association with classification. To create a variety of decision trees in each iteration step, RF divides the input samples into two groups: randomly selected samples with replacement, the size of which is the same as the input; and remaining samples, known as out-of-bag samples, the size of which is stochastically about one-third of the input. The RF repeats the procedure that consists of the following two steps: constructing the decision tree by using the randomly selected samples and validating the tree using the out-of-bag samples.

### Identification of predictor genes for responders to FOLFOX therapy

**Figure 1:**
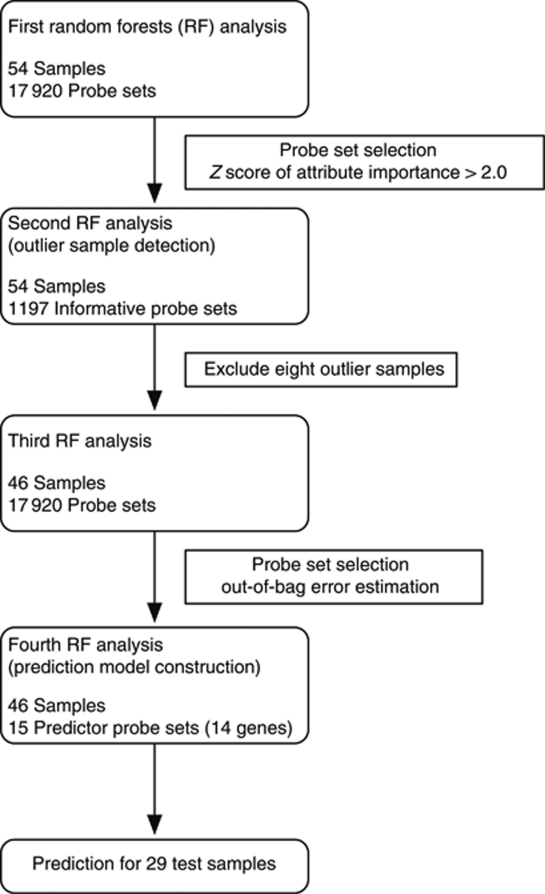
Flow diagram for the present study. Random Forests analysis was totally conducted four times. The final model with 15 probe sets was used for predicting the response of the independent 29 samples.

In the RF algorithm, the genes that confer resistance to FOLFOX therapy were ranked by frequency of occurrence in each out-of-bag cross-validation, which was repeated at 200 000 times. The top-ranking genes were selected as predictors to maximise out-of-bag classification accuracy. Using the genes selected at above step, we conduct a second RF procedure in the training set. Random Forests is not only a learning algorithm for building a class prediction model, but it calculates useful characteristics about samples, such as outlier measurements for each sample or proximity matrixes representing the similarity between all pairs of samples. For subsequent analyses, we used the proximities obtained by a second RF analysis (Figure 1).

In addition to the author (ST), two bioinformaticians independently analysed the data for reproducibility using the same algorithm.

## Results

### Assessment of clinical response by FOLFOX therapy

The training set was evaluated by computed tomography after four cycles of mFOLFOX6 therapy; CR was achieved in 2 patients (3.7%), and confirmed PR was seen in 25 patients (46.2%). On the other hand, disease remained stable in 15 patients (SD, 27.7%) and 12 patients (22.2%) showed PD. In the test set, a total of 15 patients (51.7%) demonstrated PR, 5 patients (17.2%) showed SD, and 9 patients (31.0%) showed PD. As cancer cells in SD patients are not sensitive due to activation molecules or pathways for drug resistance, SD patients were biologically classified as non-responders in this study. Therefore, the overall clinical response rate for mFOLFOX6 therapy was 50.6% (*n*=42), which is consistent with results from larger randomised studies using FOLFOX therapy for unresectable CRC (de Gramont *et al*, 2000; Giacchetti *et al*, 2000). Representative images are shown in Supplementary Figure S1.

On univariate analysis including clinical and pathological characteristics, no variables were significantly associated with response to chemotherapy (Table 1).

### Gene-expression profiles that reflect and predict response to FOLFOX therapy using Random Forests analysis

Gene-expression data were generated in all the cancer samples followed by mFOLFOX6 therapy. After normalisation by PLIER and filtering of expression score, 17 920 probes were analysed (Therneau and Ballman, 2008). The RF algorithm was then applied in the training set and 1197 informative probe sets were selected in order to distinguish responders from non-responders to FOLFOX therapy (Figure 1).

**Figure 2:**
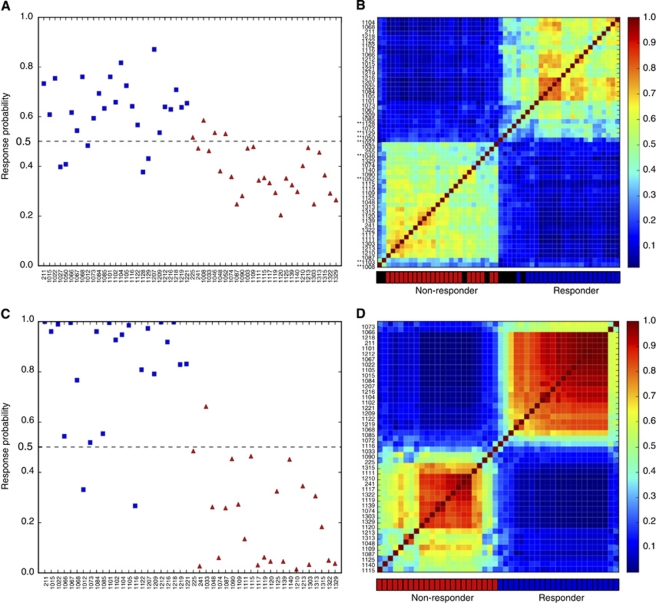
Classification accuracy of responders to FOLFOX therapy. (**A** and **B**) Classification accuracy of responders to FOLFOX therapy using 50 top-ranked genes selected by Random Forests in the training set. (**A**) Probabilities of sensitivity for FOLFOX therapy in out-of-bag cross-validation. Cutoff value was defined as response rate, 0.5. In all, 22 of 27 sensitive patients (81.4% sensitivity) and 23 of 27 resistant patients (85.1% specificity) were correctly classified, with an accuracy of 62.1% (blue square, responder; red triangle, non-responder). (**B**) Proximity matrix by predictor genes for FOLFOX therapy. At the intersection of each column and row in the figure is a pixel, the intensity of which is a measure of the distance (defined as 1−Peason's correlation coefficient) between the centroids named by the intersecting column and row. The red area corresponds to a high degree of co-occurrence, that is, these samples tend to cluster in all clustering runs. Asterisk on the patient numbers indicate outliers. Red, blue, and black boxes below the proximity matrix represent non-responder, responder, and outlier, respectively. (**C** and **D**) Classification accuracy of responders for FOLFOX therapy using 15 predictor probes (14 genes) after removing 8 outliers from the training set. (**C**) Probabilities of sensitivity for FOLFOX therapy in out-of-bag cross-validation after removing 8 outliers. Sensitivity (91.3%), specificity (95.6%), and out-of-bag classification accuracy (80.2%) were markedly improved. (**D**) Proximity matrix by predictor genes for FOLFOX therapy after removing 8 outliers. Outlier scores were calculated again in 46 samples, all of which were <6.0.

Using a cutoff of 0.5, which was same as the response rate, 22 of 27 responders (81.4% sensitivity) and 23 of 27 non-responders (85.1% specificity) were correctly identified (Figure 2A). Applying a Kolmogorov–Smirnov test for statistical significance demonstrated the capacity of the predictor to stratify the patient population into two groups according to expression data (*P*<10^−5^).

### Proximity matrix based on predictor genes

The proximities that form the N × N matrix, where N indicates sample size, is one of the most useful measures to understand sample variability. To visualise and interpret the results, we formed a proximity matrix. Identification of CRC subtypes by proximity matrix is shown in Figure 2B with three subtypes corresponding to responders, non-responders and unclassified clusters. According to the RF website (http://www.stat.berkeley.edu/ ~breiman/RandomForests/), outlier values are defined as follows:



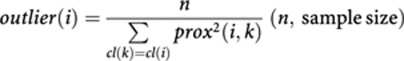



Patients are classified as outliers when the outlier value is more than 6.0; 23 patients were classified as responders, 23 patients as non-responders, and 8 patients as outliers.

Thus, the proximity matrix gives robust and reproducible clustering, and enables us to classify responders and non-responders and to extract outliers, which is clinically valuable for selection of candidates for FOLFOX therapy.

### Confirmation of predictor genes based on proximity in validation samples

Outliers identified by proximity matrix may adversely affect the selection of predictor genes in RF. To identify predictor genes that better stratify patients, the RF algorithm was applied to the remaining 46 training samples after removing the 8 outlier samples from the 54 training samples (Figure 2C). The top-ranked 50 predictor genes are listed in Table 2. To determine an appropriate number of genes that can predict resnponders to FOLFOX therapy more accurately, out-of-bag classification accuracy was calculated using various top-ranked genes. If several probes corresponding the same gene appeared repeatedly, only one representative probe was selected. Out-of-bag classification accuracy was maximised when the top 15 probes (14 genes) were applied to RF analysis (Figure 1). Using these 14 classifier genes (*SMURF2, MBTD1, AP3M2, RNF141, NPEPPS, BPTF, FAM73A, APPBP2, AMZ2P1, SRGAP1, NMT1, CSPP1, EIF1,* and *CEP290*), RF correctly classified 21 of 23 responders (91.3% sensitivity) and 22 of 23 non-responders (95.6% specificity), and achieved 80.2% out-of-bag classification accuracy (Figure 2D).

**Figure 3:**
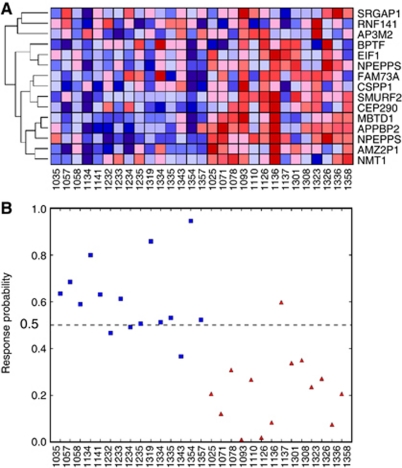
Predicted probabilities using 14 predictor genes for FOLFOX therapy in test samples. Using the prediction model in the training set, 12 of 15 sensitive patients (80.0% sensitivity) and 13 of 14 resistant patients (92.8% specificity) were correctly classified, with an averaged accuracy rate of 69.2% in the test set. The order of samples in **A** correspond to the **B**. (**A**) The heat map of the expression values of 14 predictor genes. As NPEPPS has two probes, the heat map has 15 rows. (**B**) Predicted response probability of 29 test samples (blue square, responder; red triangle, non-responder).

Based on a cutoff 0.50, as defined in the training set, such predictor genes worked well to predict response to FOLFOX therapy within the 29 independent validation samples (Figure 1), that is, correctly classfying 12 of 15 (80.0%) responders and 13 of 14 (92.8%) non-responders with an accuracy rate of 69.2% (Figure 3B). The 14 predictor genes were up-regulated in the non-responder groups, and the samples of which the predictor genes were strongly up-regulated tended to be predicted as non-responder with more certainty, that is, low response probability, and *vice versa* (Figure 3A).

To confirm the robustness of the 14 selected predictor genes, we conducted additional analyses, in which 83 samples were randomly divided five times into 54 training and 29 test samples. Consequently, we were able to classify the samples with high accuracy (Supplementary Table S3), and the same 5 genes (*SMURF2, MBTD1, NPEPPS, APPBP2,* and *AMZ2P1*) among the 14 predictors were selected as predictors in these additional analyses.

### Prediction of overall survival by response signature to FOLFOX therapy

In order to confirm whether patients who were identified as responders had a survival advantage when compared with non-responders, the survival characteristics of the two patient populations were examined.

**Figure 4:**
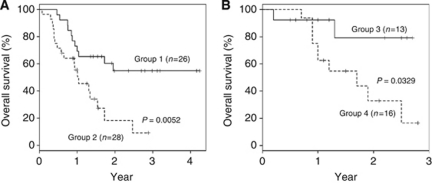
Overall survival of unresectable colorectal cancer patients. The response signature was used to predict overall survival in a training set (**A**) and a test set (**B**) of unresectable CRC patients treated with FOLFOX therapy. The predicted probability of the signature was used to identify individual patients exhibiting the phenotype. Continuous line, patients determined as responders by Random Forests algorithm; broken line, non-responders.

In out-of-bag cross-validation of the training set, 26 patients were identified determined as responders to FOLFOX therapy by RF (Group 1), and 28 patients were classified non-responders (Group 2). The survival characteristics of these patients were then examined by Kaplan–Meier survival analysis (Figure 4A). Patients in Groups 1 and 2 had median survival times of 35.1 and 12.5 months, respectively. Given these results, despite other therapies after FOLFOX in Group 2 patients, the survival distributions of the patients corresponding to the two groups were significantly different (*P*=0.0052).

On the other hand, in the test set, 13 patients were predicted to be responders to FOLFOX therapy by RF (Group 3), and 16 patients were classified as non-responders (Group 4). Figure 4B shows the Kaplan–Meier survival analysis. The 2-year overall survival rates in the patients in Groups 3 and 4 were 69.8% and 38.6%, respectively. Group 3 had a significantly longer overall survival than Group 4. Thus, the response signature to FOLFOX therapy is a useful prognostic factor for unresectable CRC outcome.

## Discussion

Treatment of unresectable CRC is empirical and most CRC patients are candidates for FOLFOX therapy as a first-line treatment. Consistent with the literature (de Gramont *et al*, 2000; Giacchetti *et al*, 2000), our data show that only half of patients benefit from FOLFOX therapy, indicating that a significant fraction of patients should undergo other treatments. Clinical and pathological data may not be available to sufficiently stratify such patients, whereas genetic approaches have been demonstrated to be effective for this purpose (Hess *et al*, 2006; Del Rio *et al*, 2007; Dressman *et al*, 2007). In this study, we developed a multigene predictor of responders to FOLFOX therapy using gene-expression signature, which may predict both tumour response and overall survival of patients.

In order to determine the gene signature for response prediction from microarray analysis, many researchers have tried to develop and apply the most accurate classification algorithms. Among such algorithms, SVM and RF are the predominant statistical methods and comparison of these algorithms for microarray-based classification has been reported (Wu *et al*, 2003; Diaz-Uriarte and Alvarez de Andres, 2006; Statnikov *et al*, 2008; Kim *et al*, 2009). In this study, we assumed that RF is a more appropriate algorithm than SVM for the following reasons: first, RF is applicable when there are more predictors than observations (Breiman, 2001); and second, RF consists of many decision trees, which is better suited to predicting response to multiagent chemotherapy. Random Forests also performs embedded gene selection and is relatively insensitive to large numbers of irrelevant genes, which enabled us to identify 14 predictor genes.

Hierarchical clustering analysis has been one of the most successful tools for displaying microarray expression data. However, its results are sensitive to outliers and this makes it difficult to assess the significance of results. On the other hand, proximity matrix, which is an important part of the RF imputation method that includes pairwise similarities between all pairs of probands, gives a robust and reproducible clustering, capable of capturing small signal variations (Breiman, 2001). In addition, proximity matrix can identify outlier samples whose proximities to all other cases in the data are generally small. In the present study, two-way hierarchical clustering analysis failed to significantly separate responders and non-responders (data not shown). Meanwhile, applying proximity matrix to data by RF algorithm, it was possible to separate responders, non-responders, and outliers, which were consistent with visualisation by two-dimensional eigenvalue decomposition analysis (data not shown). After removing outliers from training samples, we were able to identify more predominant classifier genes by which independent validation samples were correctly classified with higher accuracy.

Among the selected predictor genes in this study, several cisplatin-related genes were included. *SMURF2*, a member of the HECT family of E3 ubiquitin ligases, is thought to regulate the expression of Smad2 through a ubiquitination- and proteasome-dependent degradation process during TGF*β* signalling (Lin *et al*, 2000; Zhang *et al*, 2001). On the other hand, several researchers have reported that TGF*β* increases sensitivity to cisplatin for cancer treatment (Thavaraj *et al*, 2005; Irigoyen *et al*, 2010) and therefore, *SMURF2* will be available as a biomarker for identification of responders for FOLFOX therapy.

The increased *ANKRD40* mRNA transcript in non-responders to FOLFOX therapy is intriguing. Ankyrin repeats is a short motif that mediates protein–protein interactions and found in proteins of diverse function, and ankyrin-repeat proteins such as p16 (Tang *et al*, 2003) and Notch proteins (Aster *et al*, 2000) have been associated with cancer. Furthermore, up-regulation of *ANKRD1* was associated with decreased sensitivity for cisplatin in ovarian adenocarcinoma (Scurr *et al*, 2008). In addition to our data, ankyrin-repeat domains may enhance platinum resistance.

Other than cisplatin-resistant genes, genes including *CIRBP* (Zeng *et al*, 2009) and *CREB1* (Shukla *et al*, 2009), the over-expression of which is reported in chemotherapy-resistant cells, were also up-regulated in non-responders in the present study.

In summary, RF analysis for multiagent therapy not only identified predictor genes, but also stratified patients by tumour response, independently of established clinicopathological variables. We believe that the present approach is effective for predicting responders to chemotherapy, and will be useful as a first step in establishing personalised therapy.

## Figures and Tables

**Table 1:** Clinical and pathological characteristics of patients

	**Responder (*n*=42)**	**Non-responder (*n*=41)**	**Total (*n*=83)**	***P*-value**
Age (years)	64.0±10.2	61.8±12.3	62.9±11.3	NS^a^
Gender (male/female)	27/15	27/14	54/29	NS^b^
CEA (ng ml^−1^)	299.0±772.9	339.9±756.6	319.2±760.5	NS^c^
CA19-9 (U ml^−1^)	418.6±941.6	536.4±1085.2	476.8±1010.6	NS^c^
Differentiation grade (well/mod/por)	32/9/1	30/7/4	62/16/5	NS^b^
Primary lesion (rt/lt)	18/24	18/23	36/47	NS^b^
Metastatic lesion (liver/lung/bone/peritoneum)	32/7/0/3	32/3/2/4	64/10/2/7	NS^b^

Abbreviations: CA19-9=carbohydrate antigen 19-9; CEA=carcinoembryonic antigen; lt=left; mod=moderately; NS=not significant; por=poorly; rt = right.

a *t*-test.

b Fisher's test.

c Wilcoxon's test.

**Table 2:** Top 50 classifier genes after exclusion of outliers

**Probe set ID**	**Gene symbol**
227489_at	SMURF2
226797_at	MBTD1
203410_at	AP3M2
226106_at	RNF141
214101_s_at	NPEPPS
231953_at	BPTF
201455_s_at	NPEPPS
235125_x_at	FAM73A
202630_at	APPBP2
223443_s_at	AMZ2P1
1555875_at	SRGAP1
201158_at	NMT1
227105_at	CSPP1
217672_x_at	EIF1
205250_s_at	CEP290
202880_s_at	CYTH1
1552283_s_at	ZDHHC11
225384_at	DOCK7
202034_x_at	RB1CC1
222715_s_at	SYNRG
226441_at	MAP3K2
240304_s_at	**TMC5**
200090_at	FNTA
53071_s_at	C17orf101
212561_at	DENND5A
200811_at	CIRBP
206600_s_at	SLC16A5
227064_at	ANKRD40
205596_s_at	SMURF2
230211_at	–
203651_at	ZFYVE16
226580_at	BRMS1L
222589_at	NLK
227564_at	HGSNAT
64418_at	SYNRG
223595_at	TMEM133
230621_at	IAH1
225572_at	CREB1
225198_at	VAPA
202460_s_at	LPIN2
212704_at	ZCCHC11
204485_s_at	TOM1L1
212397_at	RDX
225595_at	CREBZF
202814_s_at	HEXIM1
204208_at	RNGTT
222656_at	UBE2W
203116_s_at	FECH
227395_at	FLJ38498
201454_s_at	NPEPPS

Bold entry: down-regulated genes in non-responders.
