# Supplementary material for: Potential responders to FOLFOX therapy for colorectal cancer by Random Forests analysis
Source: Br J Cancer. 2011 Nov 17;106(1):126–32. doi: 10.1038/bjc.2011.505 (PMC3251854; doi:10.1038/bjc.2011.505)
Supplement: Supplementary Information [file bjc2011505x1.doc]

Supplementary Online Material for

**Potential responders to FOLFOX therapy for colorectal cancer by Random Forests analysis**

Shingo Tsuji, Yutaka Midorikawa, Takao Takahashi, Koichi Yagi, Tadatoshi Takayama, Kazuhiro Yoshida, Yasuyuki Sugiyama* and Hiroyuki Aburatani

***Correspondence:** Yasuyuki Sugiyama

E-mail: ysugi@med.teikyo-u.ac.jp

This file includes Supplementary Table S1, S2, S3, and Supplementary Figure S1.

| Supplementary Table S1 Clinicopathological data according to drug response in the training set | | | | | | | | |
| --- | --- | --- | --- | --- | --- | --- | --- | --- |
| sample no. | age | gender | location | Pr/Meta | CEA (ng/ml) | CA19-9 (U/ml) | diff. grade | response |
| *responder* |  |  |  |  |  |  |  |  |
| 211 | 54 | F | Liver | Meta | 36 | 57 | well | PR |
| 1015 | 55 | M | Rectum | Pr | 7 | 4 | well | PR |
| 1022 | 71 | M | Descending | Pr | 17 | 6 | well | PR |
| 1027 | 73 | M | Transverse | Pr | 16 | 12 | well | PR |
| 1050 | 78 | M | Rectum | Pr | 88 | 3 | well | PR |
| 1066 | 84 | F | Cecum | Pr | 29 | 37 | well | PR |
| 1067 | 55 | M | Ascending | Pr | 3 | 358 | mod | PR |
| 1068 | 70 | M | Rectum | Pr | 14 | 21 | well | PR |
| 1072 | 55 | M | Rectum | Pr | 237 | 353 | well | PR |
| 1073 | 58 | M | Sigmoid | Pr | 3990 | 330 | mod | PR |
| 1084 | 74 | M | Sigmoid | Pr | 521 | 274 | well | PR |
| 1085 | 66 | M | Rectum | Pr | 962 | 1440 | mod | PR |
| 1101 | 59 | M | Peritoneum | Meta | 38 | 1620 | well | PR |
| 1102 | 62 | M | Liver | Meta | 231 | 238 | well | PR |
| 1104 | 55 | M | Liver | Meta | 8 | 5 | well | PR |
| 1105 | 70 | M | Peritoneum | Meta | 12 | 16 | well | CR |
| 1116 | 55 | M | Peritoneum | Meta | 3 | 358 | mod | PR |
| 1122 | 55 | F | Liver | Meta | 223 | 115 | mod | PR |
| 1128 | 71 | M | Liver | Meta | 12 | 11 | well | PR |
| 1129 | 55 | F | Liver | Meta | 4 | 13 | well | CR |
| 1207 | 70 | M | Rectum | Pr | 31 | 345 | mod | PR |
| 1209 | 41 | M | Ascending | Pr | 54 | 13 | well | PR |
| 1212 | 56 | M | Ascending | Pr | 30 | 21 | por | PR |
| 1216 | 68 | M | Sigmoid | Pr | 609 | 7 | mod | PR |
| 1218 | 76 | F | Rectum | Pr | 6 | 57 | mod | PR |
| 1219 | 54 | F | Rectum | Pr | 3 | 91 | well | PR |
| 1221 | 77 | F | Ascending | Pr | 6 | 206 | well | PR |
|  |  |  |  |  |  |  |  |  |
| *non-responder* |  |  |  |  |  |  |  |  |
| 225 | 68 | F | Liver | Meta | 56 | 19 | well | PD |
| 241 | 50 | M | Liver | Meta | 676 | 720 | well | PD |
| 1008 | 63 | M | Rectum | Pr | 168 | 169 | well | PD |
| 1033 | 80 | M | Cecum | Pr | 4 | 1 | well | SD |
| 1046 | 71 | M | Ascending | Pr | 1650 | 2 | well | SD |
| 1048 | 74 | M | Ascending | Pr | 66 | 1 | well | PD |
| 1052 | 70 | M | Ascending | Pr | 8 | 25 | well | SD |
| 1074 | 53 | M | Rectum | Pr | 2 | 385 | mod | SD |
| 1087 | 61 | F | Rectum | Pr | 2780 | 2830 | mod | SD |
| 1090 | 71 | M | Rectum | Pr | 13 | 37 | well | SD |
| 1103 | 63 | M | Liver | Meta | 199 | 1 | well | PD |
| 1109 | 38 | M | Liver | Meta | 249 | 422 | well | SD |
| 1111 | 59 | M | Liver | Meta | 198 | 1 | well | SD |
| 1115 | 71 | M | Lung | Meta | 16 | 15 | well | SD |
| 1117 | 74 | M | Liver | Meta | 179 | 1 | well | PD |
| 1119 | 73 | M | Liver | Meta | 3 | 13 | well | PD |
| 1120 | 57 | F | Liver | Meta | 15 | 40 | por | SD |
| 1125 | 71 | F | Liver | Meta | 70 | 4760 | well | SD |
| 1139 | 31 | M | Liver | Meta | 3 | 3311 | well | PD |
| 1140 | 65 | M | Liver | Meta | 346 | 553 | well | PD |
| 1210 | 44 | F | Sigmoid | Pr | 1609 | 2146 | mod | SD |
| 1213 | 59 | M | Rectum | Pr | 87 | 1 | mod | SD |
| 1303 | 72 | F | Sigmoid | Pr | 157 | 813 | well | PD |
| 1313 | 66 | M | Sigmoid | Pr | 23 | 15 | well | SD |
| 1315 | 55 | F | Sigmoid | Pr | 5 | 9 | well | SD |
| 1322 | 59 | M | Rectum | Pr | 781 | 81 | well | PD |
| 1329 | 69 | M | Rectum | Pr | 2 | 590 | por | PD |
| M, male; F, female; Pr, primary colorectal cancer; Meta, metastatic colorectal cancer; diff. grade, differentiation grade of the primary colorectal cancer; CR, complete response; PR, partial response; SD, stable disease; PD, progressive disease. | | | | | | | | |

| Supplementary Table S2 Clinicopathological data according to drug response in the test set | | | | | | | | |
| --- | --- | --- | --- | --- | --- | --- | --- | --- |
| sample no. | age | gender | location | Pr/Meta | CEA (ng/ml) | CA19-9 (U/ml) | diff. grade | response |
| *responder* |  |  |  |  |  |  |  |  |
| 1035 | 68 | F | Rectum | Pr | 19 | 111 | well | PR |
| 1057 | 70 | M | Sigmoid | Pr | 238 | 4500 | well | PR |
| 1058 | 65 | F | Sigmoid | Pr | 3 | 1 | well | PR |
| 1134 | 59 | F | Liver | Meta | 2590 | 363 | well | PR |
| 1141 | 77 | M | Liver | Meta | 26 | 48 | well | PR |
| 1232 | 55 | M | Sigmoid | Pr | 92 | 1 | well | PR |
| 1233 | 60 | M | Descending | Pr | 5 | 224 | well | PR |
| 1234 | 55 | M | Rectum | Pr | 286 | 1031 | well | PR |
| 1235 | 69 | M | Rectum | Pr | 1929 | 514 | well | PR |
| 1319 | 71 | F | Rectum | Pr | 35 | 84 | well | PR |
| 1334 | 39 | F | Sigmoid | Pr | 10 | 357 | well | PR |
| 1335 | 77 | F | Rectum | Pr | 39 | 44 | well | PR |
| 1343 | 59 | F | Sigmoid | Pr | 25 | 36 | well | PR |
| 1354 | 72 | M | Cecum | Pr | 7 | 229 | mod | PR |
| 1357 | 79 | F | Ascending | Pr | 64 | 4030 | well | PR |
| *non-responder* |  |  |  |  |  |  |  |  |
| 1025 | 44 | F | Sigmoid | Pr | 74 | 9 | well | PD |
| 1071 | 73 | M | Sigmoid | Pr | 4 | 1250 | well | SD |
| 1078 | 75 | F | Rectum | Pr | 2 | 2950 | well | SD |
| 1093 | 74 | M | Rectum | Pr | 90 | 19 | mod | SD |
| 1110 | 55 | M | Liver | Meta | 9 | 58 | well | PD |
| 1126 | 59 | M | Liver | Meta | 3530 | 15 | well | PD |
| 1136 | 65 | F | Liver | Meta | 6 | 10 | well | PD |
| 1137 | 36 | F | Liver | Meta | 2 | 201 | well | PD |
| 1301 | 35 | F | Rectum | Pr | 1 | 7 | mod | PD |
| 1308 | 59 | M | Sigmoid | Pr | 830 | 5 | mod | SD |
| 1323 | 72 | F | Ascending | Pr | 5 | 388 | well | PD |
| 1326 | 65 | F | Cecum | Pr | 14 | 17 | por | PD |
| 1336 | 59 | M | Sigmoid | Pr | 2 | 6 | por | SD |
| 1358 | 76 | M | Ascending | Pr | 2 | 97 | well | PD |
| M, male; F, female; Pr, primary colorectal cancer; Meta, metastatic colorectal cancer; differentiation grade on the primary colorectal cancer; CR, complete response; PR, partial response; SD, stable disease; PD, progressive disease. | | | | | | | | |

| Supplementary Table S3 Summary of the results of additional analysis | | | | | |
| --- | --- | --- | --- | --- | --- |
|  | Set1 | Set2 | Set3 | Set4 | Set5 |
| Sensitivity | 80.0 | 73.3 | 86.7 | 73.3 | 86.7 |
| Spesificity | 78.6 | 100.0 | 78.6 | 92.9 | 64.3 |
| Accuracy | 69.0 | 79.9 | 62.9 | 70.4 | 71.3 |
| Number of predictor genes | 15 | 7 | 14 | 14 | 8 |
| The 83 samples were randomly divided into 54 training samples and 29 validation samples, then the data set were analyzed by Random Forests procedures. The table indicates the results summary of five different validation data sets. | | | | | |


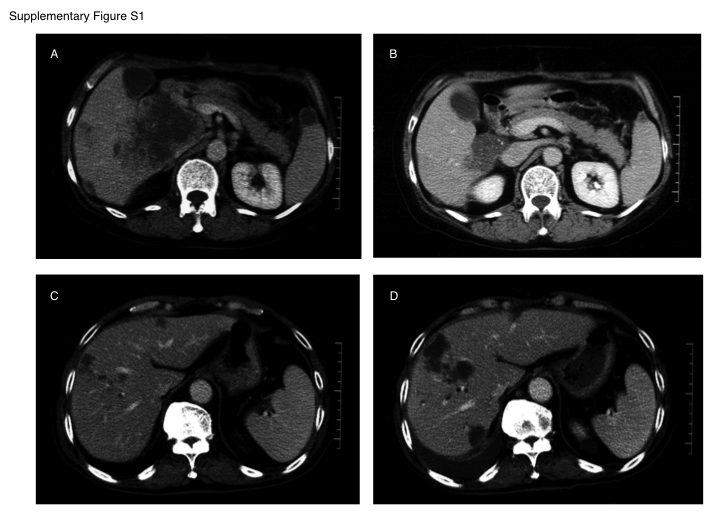


Assessment of response to mFOLFOX6 therapy by computed tomography.

A, B) Responder before (A) and after (B) therapy

C, D) Non-responder before (C) and after (D) therapy
